# Determination of the Sampler Type and Rainfall Effect on the Deposition Fluxes of the Polychlorinated Biphenyls

**DOI:** 10.1100/2012/798020

**Published:** 2012-05-02

**Authors:** Askin Birgül, Yücel Tasdemir

**Affiliations:** Department of Environmental Engineering, Engineering and Architecture Faculty, Uludag University, Gorukle 16059, Bursa, Turkey

## Abstract

Atmospheric concentration and deposition samples were collected between June 2008 and June 2009 in an urban sampling site Yavuzselim, Turkey. Eighty-three polychlorinated biphenyl (PCB) congeners were targeted in the collected samples. It was found that 90% of the total PCB concentration was in the gas phase. Deposition samples were collected by a wet-dry deposition sampler (WDDS) and a bulk deposition sampler (BDS). Average total deposition fluxes measured with the BDS in dry periods was 5500 ± 2400 pg/(m^2^day); average dry deposition fluxes measured by the WDDS in the same period were 6400 ± 3300 pg/(m^2^day). The results indicated that the sampler type affected the measured flux values. Bulk deposition samples were also collected in rainy periods by using the BDS and the average flux value was 8700 ± 3100 pg/(m^2^day). The measured flux values were lower than the values reported for the urban and industrial areas. Dry deposition velocities for the WDDS and BDS samples were calculated 0.48 ± 0.35 cm/s and 0.13 ± 0.15 cm/s, respectively.

## 1. Introduction

Their widespread use and properties have led PCBs to become globally distributed. Since production began in the 1930s, approximately 1.3 million tonnes of PCB have been manufactured and used in numerous applications, for example, as coolants and insulating fluids for transformers and capacitors, stabilizing additives in PVC coatings, pesticide extenders, cutting oils, flame retardants, hydraulic fluids, sealants, adhesives, wood floor finishes, carbonless copy paper, and paints [1, 2].

PCBs may enter the atmosphere from a variety of diffuse sources, such as leakage of PCB-containing electrical installations (capacitors and transformers) that are still in use or stored at landfills, combustion of municipal and industrial wastes, or volatilization from contaminated buildings [3, 4]. The food chain is the main source of human exposure to PCBs.

Urban and industrial areas are major sources of atmospheric PCBs to surrounding regions [5]. Atmospheric transport from major urban industrial areas can lead to a significant PCB loading to neighbouring terrestrial [6] and aquatic ecosystems [7], by diffusive air-water exchange, air-vegetation exchange, wet deposition (rain-snow), and dry particle deposition. Once delivered, PCBs may be remobilized to the regional atmosphere by air-surface exchange processes [8, 9].

Atmospheric deposition is an important source of organic and inorganic contamination, consequently many studies have been performed by researchers in recent years to estimate the deposition values [10–13]. Various techniques such as Teflon surfaces, petri plates, water surfaces, and greased surfaces have been applied to determine atmospheric dry deposition fluxes of semivolatile organic compounds (SVOCs) [14–17]. Bulk deposition is achieved with a sampler which is always open to the atmosphere, thus wet-and-dry deposition takes place simultaneously [13].

Bursa (40°10′58.17′′N, 29°4′6.32′′E) is the 4th biggest city of Turkey located in the northwest of Marmara region with a population of 2.5 million people. It is an important transportation route and many industrial districts have been established in Bursa. PCBs have been measured in different environmental compartments around the world, but measurements in air are limited in Turkey. In order to assess this, potential of priority organic pollutants, gas-particle concentrations, temporal changes of dry and bulk deposition fluxes, and dry and bulk deposition velocities of these compounds were determined in urban air of Bursa, Turkey. This paper reports some of that work, focussing on a comparison of deposition samplers and derivation of deposition flux information.

## 2. Materials and Methods

### 2.1. Sampling Program

Thirty-four ambient air samples and 23 dry deposition and bulk deposition samples were collected from Yavuzselim (YS) sampling site between June 2008 and June 2009, in order to determine dry deposition and bulk deposition fluxes associated with atmospheric concentrations of PCBs.

YS was a residential site (40°11′48.40′′N-29°5′46.80′′E) and located about 500 m away from the nearest major road. The sampling site was within the boundaries of Yıldırım Municipality and the samplers were placed on the roof of a 3-storey building. The YS sampling site was surrounded by residences and small workplaces and in this region natural gas and coal were mainly used as a fuel.

At the sampling site, one high volume air sampler (HVAS) (glass fibre filter (GFF) of 90 mm outer diameter (o.d.) and pore size of 1.6 *μ*m, polyurethane foam (PUF) plug 50 mm high × 65 mm length (o.d) and density of 0.0225 g/cm^3^, GPS 11, Thermo Andersen Inc., USA) and one wet and dry deposition sampler (WDDS) (each sampling part 40 × 40 cm (0.16 m^2^) and a depth of 70 cm, TYN 400, Teknosem, Turkey) were deployed at the sampling site.

The HVAS was calibrated using a standardized orifice manometer kit (Thermo Andersen Inc., USA) based on the manufacturers requirements for calibration. The mean flow rate and the sampling volume for each sample were about 0.20 m^3^/min and 260 m^3^, respectively. The flow rates were checked before and after sampling by calibrated flow meters. Both gaseous and particulate phase PCBs were collected over four seasons, namely, from June 2008 to June 2009. There was a meteorological station in the sampling site in order to provide the meteorological data (Davis Vantage Pro2, Davis Instruments Corp., USA). The meteorological data recorded during the sampling period are summarized in Table 1.

### 2.2. Sample Collection

Ambient air samples were collected by means of the HVAS when there was no rainfall. Deposition samples (both dry and wet) were collected by a WDDS which was modified by our research group. The WDDS was manufactured from stainless steel and composed of two parts. The first part was the dry deposition section where dry deposition samples were collected in the periods when there was no rain and second part was the wet deposition section where rain samples were collected in the rainy periods. There was an active cover on the device which operated after a signal was taken from the rain sensor. After rain stopped, the cover opened on top of the dry deposition part and closed on top of the wet deposition part. Details of the WDDS have been previously described elsewhere [17, 18]. Samples were taken for 15-day periods.

**Figure 1:**
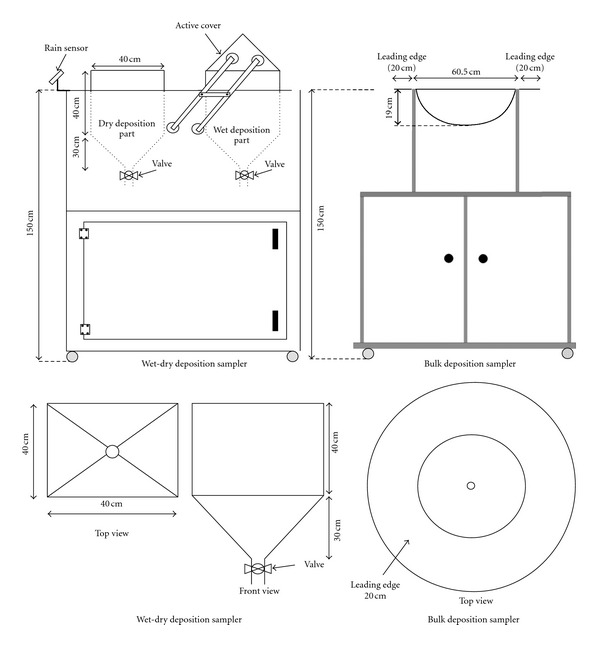
Schematic display of wet dry deposition sampler (WDDS) and bulk deposition sampler (BDS).

Bulk deposition samples were collected with bulk deposition samplers (BDSS) manufactured from stainless steel with a diameter of 60.5 cm and a depth of 19 cm. There was a leading edge with a width of 20 cm in order to prevent negative effects of turbulence around the BDS [18]. The BDS and WDDS were run simultaneously. Schematic views of the WDDS and BDS are shown in Figure 1.

### 2.3. Analytical Procedure

Prior to extraction, the PUF, GFF and XAD-2 resin samples were spiked with a surrogate standard consisting of PCB 14, PCB 65, and PCB 166 (4 ng/mL each) to determine the analytical recoveries of the PCBs. The sampling, extraction, and analysis procedures followed in this study were explained elsewhere and were only summarized here [17, 18]. Briefly, PUF cartridges were soxhlet extracted with a 1 : 4 (v : v) mixture of DCM/PE (petroleum ether) for 24 hours [19]. HVAS filters were extracted with 25 mL DCM/PE (dichloromethane/petroleum ether, 1 : 4) mixture for 30 minutes in an ultrasonic bath (S80H, Elma GmbH Co., Germany). This step was repeated twice. Then, the bottle containing the sample was rinsed with the same 5 mL solvent mixture and added to other solvent mixture.

Before the collection of WDDS samples, the dry side was wiped clean with a paper napkin using ACE/HEX (acetone/hexane, 1 : 1) mixture. At the end of the sampling period, the dry deposition side of the WDDS was again cleaned with a paper napkin and then ACE/HEX (1 : 1) was rinsed and finally was wiped with the paper napkin again. The paper napkins and ACE/HEX (1 : 1) mixture used in rinsing were kept in Teflon-coated jar. These dry deposition samples were extracted with 100 mL ACE/HEX (1 : 1) mixture in an ultrasonic bath for 30 minutes twice.

 If there was water on the sampler due to rainfall, this was filtered through XAD-2 resin. The resin was then extracted with 100 mL ACE/HEX (1 : 1) mixture for 30 minutes with an ultrasonic bath. This process was repeated once more with another 100 mL ACE/HEX mixture. Bulk deposition samples were filtered through from sodium sulphate (Na_2_SO_4_) after extraction in order to remove any residual water in the samples. The BDS surface was then rinsed with ACE/HEX mixture. This process was repeated a few times and the solvents were stored in a jar. Finally, the BDS surface was wiped with a paper napkin to remove contaminants from the surface and the used paper napkins were put into sample bottle so as to analyze them.

The volume of the extracted samples was reduced to 2 mL with the use of rotary evaporator and gentle stream of nitrogen (N_2_), and these samples were passed through a cleanup column including 1 cm sodium sulphate (Na_2_SO_4_), 2 g of aluminium oxide (120 *μ*L pure water to 2 g of aluminium oxide), and 3 g of silica (100 *μ*L pure water to 3 g of silica). The column was cleaned with 20 mL DCM and then with 25 mL PE. The volume of the PCB samples in PE was reduced to 5 mL, and the solvent exchange was performed by adding 15 mL HEX. This was repeated twice. Finally, samples with reduced volume of 2 mL were rinsed with acid and taken to the vials. An internal standard solution consisting of PCB 30 and PCB 204 was added to concentrated samples for volume correction and internal standard was added just before the quantification of the PCB compounds.

Quantification of PCB congeners was conducted using an Agilent 7890A model gas chromatograph equipped with a *μ*ECD (Micro-Electron Capture Detector) (Hewlett-Packard, USA). GC-ECDs have been employed for PCB analysis in many studies because of their sensitivity [6, 20–22]. The oven temperature program used in the PCB analyses was 70°C (2 min), increasing with 25°C/min to 150°C, then 3°C/min to 200°C, then 8°C/min to 280°C, followed by 8 minutes of holding under 280°C, increasing with 10°C/min to 300°C and holding for 2 minutes. The final program time was 41.87 minutes. The inlet temperature was kept at 250°C and the detector temperature was 320°C. The carrying gas was helium (He) (1.9 mL/min) and the make up gas was N_2_ (25 mL/min). HP5-MS (30 m × 0.32 mm × 0.25 *μ*m, Agilent, 19091 Je413) was used as a capillary column. For the calibration of the instrument, five levels of standard solutions ranging between 0.05 and 25 ng/mL were used for calibration. After each 25 samples injection, the medium standard was injected to check instrument stability. The instrument detection limit, IDL, was determined as 0.1 pg for 1 *μ*L injection. The linear *r* ^2^ values determined with these standards varied between 0.99457 and 0.99996 for each PCB congener.

### 2.4. Quality Control (QC)/Quality Assurance (QA)

A total of 83 PCB congeners were targeted in the collected samples: PCB#4/10, #9/7, #6, #8/5, #19, #12/13, #15/17, #16/32, #26, #31, #28, #21, #53, #22, #45, #52, #47, #49/48, #44, #37/42, #71/41/64, #100, #74, #70/61, #66/95, #91, #56/60, #92, #84, #89/101, #99, #119, #83, #81/87, #86, #85, #77/110, #135/144, #114/149, #118, #123, #131, #153, #132/105, #163/138, #126, #128, #167, #174, #202/171/156, #172, #180, #200, #170/190, #169, #199, #207, #194, #205, and #206.

In order to determine the probable contamination generated during sampling, extraction, and analysis, 10% of the total samples were taken as blank samples. Same transportation, extraction, and analysis procedure applied to the real samples were employed to the blanks.

The limit of detection (LOD) values were calculated by adding three times standard deviations of the blank samples to the average PCB concentrations in the blank samples [23–26]. LOD values were determined for each PCB congener and data smaller than the LOD values were neglected.

The ratio of the average PCB value obtained in the blank samples to the PCB values determined in the samples were found as 5.7 ± 3.5% for GFF, 1.7 ± 1.0% for PUF, 3.0 ± 2.8% for WDDS, and 3.3 ± 3.5% for the BDS. The average recovery efficiencies for the PCB 14, PCB 65, and PCB 166 were shown in Table 2. All results in this study have been reported after surrogate and internal correction.

## 3. Results and Discussions

### 3.1. Ambient Air Concentrations

**Figure 2:**
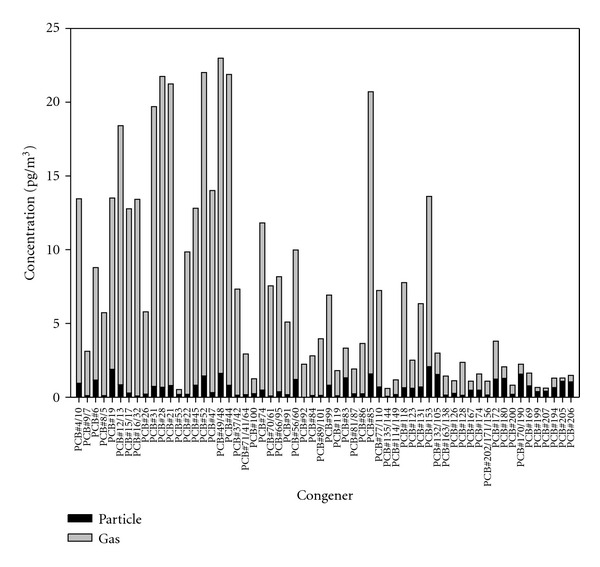
Distribution of the average gas/particulate concentrations of the PCB congeners.

During the sampling period, 34 atmospheric air samples were collected by means of the HVAS. Two samples were collected in the first half of the month and the other two were taken in the second half when there was no rain. The average gas and particulate phase PCB concentrations (mean ± SD) were 320 ± 110 pg/m^3^ and 40 ± 30 pg/m^3^, respectively. Total PCB concentration was determined to be 360 ± 100 pg/m^3^ at the YS sampling site. These levels of concentrations were among the high levels determined in urban sites (Table 3) [3, 4, 29, 31]. The high PCB levels suggested that the occurrence of PCBs was due to local sources and long-range transport. The area of Northwestern Turkey is known as an area with mid to high concentrations of PCBs and many studies have been conducted last years to describe the situation [16–18, 22, 33]. The most abundant individual congeners, in this study, were PCB-85, PCB-52, and PCB-28 with average concentrations of 23.6, 21.3, 20.8 pg/m^3^, respectively. Regarding the other congeners of the typical seven PCB-mix, they occurred in various concentrations with the following order: PCB-153 < PCB-118<PCB-101<PCB-180<PCB-138 (Figure 2).

The distribution between gas and particulate phase PCB congeners is shown in Figure 2. It can be seen that 90% of the total PCB burden was in the gas phase and lower molecular weight PCBs were more dominant in the collected samples because they partition mainly in the gas phase. The heavier PCBs (octa- and nona-CBs) were dominant in particulate phase because they tend to sorb onto the particulates in percents that are as high as 80%. Measured gas and particulate phase concentrations were higher than the rural site values but lower than some urban site values [3, 12, 24, 30, 31]. Various researchers were studied on PCBs in different regions and measured gas-particle phase levels are shown in Table 3.

PCBs usually exhibit seasonality, with summer values being higher than the winter ones due to the evaporation that takes place from different surfaces facilitated by the higher summer temperatures [12, 22, 25].

Total organic compound (TOC) levels were determined in the collected samples, because the TOC level might affect the sorption quantities of PCBs. In YS, the TOC content was measured for summer, fall, winter, and spring seasons as 12.3 ± 1.1, 3.02 ± 0.3, 3.91 ± 2.7, and 2.52 ± 0.19%, respectively. The values of TOCs were more than 10% in summer for YS sampling site. 

### 3.2. Dry and Bulk Deposition Fluxes

Samples (*n* = 23) were taken over biweekly (every 15 days) with BDS and WDDS, simultaneously. During the sampling, BDS was exposed to the atmosphere all the time while WDDS was sampling only during dry weather conditions. The average flux values of WDDS and BDS were 5500 ± 2400 pg/(m^2^day) and 7200 ± 3500 pg/(m^2^day), respectively. Dry deposition and bulk deposition flux values obtained in other studies carried out in different areas and taken from the literature are summarized in Table 4. Although it was mentioned earlier that the air concentrations of PCBs were quite high in YS site, the deposition values were lower than in past studies [3, 14, 33–36]. Comparing the present values with relevant study in the same city, the results are at the same order of magnitude [16], The parameters that generally affect the deposition fluxes are the sampler type, the atmospheric PCB concentrations, the sampling site characteristics, and the sampling period.

**Figure 3:**
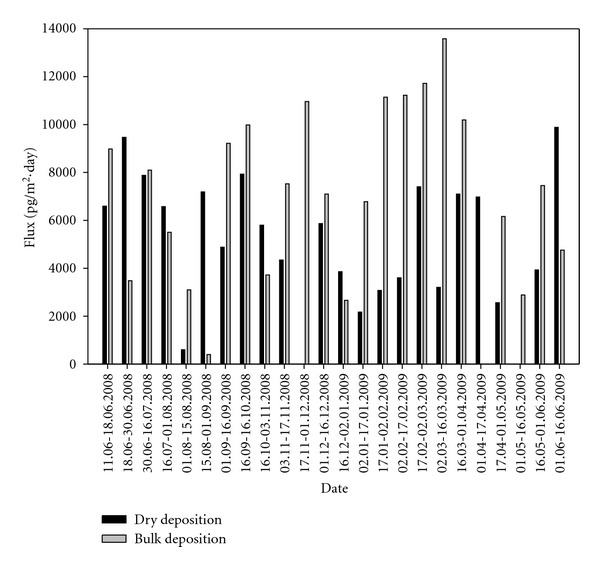
Temporal variation of dry and bulk deposition flux values.

Samples of BDS and WDDS taken only during dry periods were used to compare the dry deposition fluxes. The purpose of this comparison was to determine the possible effects of sampler types on the measured fluxes. The average total deposition flux measured with the BDS in dry periods was 5500 ± 2400 pg/(m^2^day) while in the same period, the average dry deposition flux value measured with the WDDS was 6400 ± 3200 pg/(m^2^day). Temporal variations of measured flux values are illustrated in Figure 3. Results indicate that the shape of samplers considerably affected the flux obtained. Both samplers were made of stainless steel; thus, both samplers collected only particle phase PCBs. The shapes of collection surfaces caused the deposited particles to re-suspend into the atmosphere at different amounts. Particles deposited onto the BDS having shallow depth could be re-suspended more easily with the help of wind. This caused a decrease in the flux amount. On the other hand, WDDS had a deeper collection structure with a depth of about 70 cm thus losses due to resuspension were less significant. The wind speed proved to be another important factor affecting the flux ratio. For example, the *F *_WDDS_/*F *_BDS_ values determined for three different wind speeds of <1 m/s, 1–1.5 m/s and >1.5 m/s yielded ratios of 0.73, 1.52, and 1.59, respectively.

**Figure 4:**
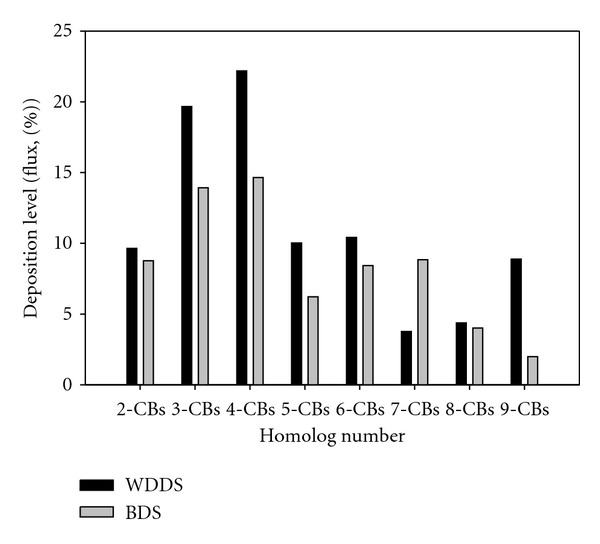
PCB homolog distribution of dry deposition flux samples.

**Figure 5:**
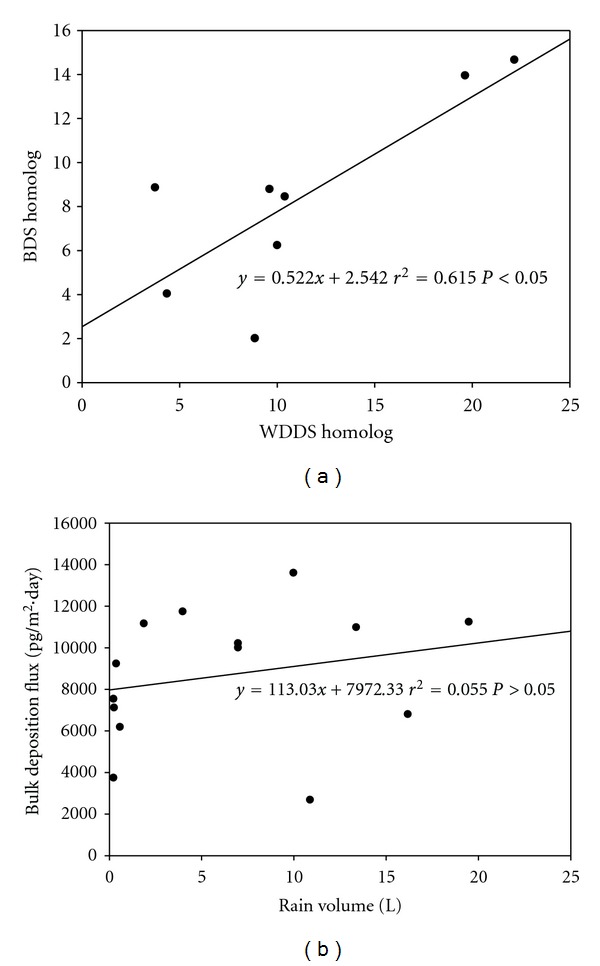
(a) Relationship between WDDS homolog distributions and BDS homolog distributions, and (b) relationship between rainy period bulk deposition fluxes and rain volumes.

When the PCB homolog group distributions of flux values which were obtained with the WDDS and BDS in the same period were analyzed, it was determined that 3-CBs and 4-CBs were dominant in this period. Homolog profiles of PCBs from both samplers for the same period are shown in Figure 4. The two profiles exhibited significant positive correlation (Figure 5(a), *r* ^2^ = 0.61, *P* < 0.05). The profiles under both sampling modes were different comparing to the ambient profile (Figure 2) for which the light PCBs were prevalent. Instead, in the deposition profiles the tetra-CBs were most abundant accounting more than 35% of the total PCBs. The reason for this fact is that the gas phase which is enriched in lighter PCBs is not deposited in the same extent as the particulate phase.

Bulk deposition samples were collected with the BDS in rainy periods, as well. Average bulk deposition flux values from rainy period samples were 8700 ± 3100 pg/(m^2^day) and were higher than the flux values obtained with the BDS in dry periods. Transport of the particulates with the rain and absorption of the gas phase PCBs into the rain drops according to Henry's law were reported in the literature for SVOCs [39–42]. These combined processes likely caused higher fluxes in rainy periods. Another reason for higher fluxes was deposited rain water on the BDS. The water on the BDS sampler captures particulates and particulates containing PCBs do not bounce off when they hit the water surface [34]. Moreover, the aqueous phase moves towards equilibrium with gas phase PCBs, transporting them to the deposition collector.

A relationship between rain volume and rainy period flux values of BDS was examined, but no statistically significant relationship was found (Figure 5(b), *P* > 0.05). However, there was a positive correlation and it indicated that deposition flux increased depending on an increase in the rain volume.

The average dry deposition flux value was 4700 ± 1900 pg/(m^2^day) for the WDDS in rainy periods. This was smaller than the value obtained with the BDS. Washout of PCBs from the atmosphere by precipitation caused a decrease of dry deposition flux values in this period.

It has been examined whether this situation resembles within the flux values. In this scope, higher dry deposition fluxes were obtained with the WDDS in hotter seasons with no rain, while higher deposition fluxes were obtained for bulk deposition fluxes in rainy season.

### 3.3. Dry and Bulk Deposition Velocities

The PCB flux values (*F*_p_) were divided by the particle phase PCB concentration (*C*_p_) values while calculating the dry deposition velocity (*V*_*d*_) values for the WDDS. This calculation was a little different for BDS. If only dry season values were calculated, this approach would be applicable for the BDS, either. On the other hand, the dry and wet depositions were observed at the same time in some samples. In these cases, flux value was divided by total concentration (gas + particle) and this new velocity can be named as bulk deposition velocity.

Dry deposition velocity values which were obtained for WDDS and BDS collected in the same periods were 0.48 ± 0.35 cm/s and 0.13 ± 0.15 cm/s, respectively. This difference was mainly caused by the wind effects as a result of the samplers shape. The deposition velocity obtained in rainy periods with BDS was 0.11 ± 0.04 cm/s. This was a little smaller than the one calculated for dry periods. This was probably because not only particulate but also gas phase deposition occurred simultaneously and the gas phase deposition velocity was lower than particulate phase [43].

It was determined that while 5-CBs, 6-CBs, and 8-CBs had higher deposition velocity in the rainy period samples of the BDS, while 7-CBs and 8-CBs had greater deposition velocities in dry period samples in which only dry deposition occurred.

It should also be noted that sampling site characteristics, atmospheric particulate matter concentrations and meteorological parameters play a crucial role on the variations of the deposition velocities.

## 4. Conclusions

Deposition fluxes and concentrations of the PCBs were measured between June 2008 and June 2009. The measured gas and particulate phase concentrations were higher than the values reported for the rural areas but lower than the values given for the urban areas.

Flux values were measured with two different samplers (WDDS and BDS). Average fluxes of WDDS in dry period were higher than the one measured with the BDS. This difference was mainly due to the shape of the sampler because both samplers were run side-by-side simultaneously. On the other hand, fluxes measured with the BDS were higher during the rainy periods. This was mainly due to scavenging of PCBs with the precipitation, capturing of the particulate phase PCBs, and absorption of gas phase PCBs into the water on the BDS. However, there was no significant relationship between rain volume and flux measured with the BDS in rainy periods.

Dry deposition velocity values calculated for the WDDS were higher when compared to dry deposition velocity values which were obtained with the BDS when there was no rainfall. Deposition velocity values obtained from the BDS differentiated depending on the dry and rainy periods. Rainy period values were lower due to the effect of gas phase. When homolog distributions of PCBs in bulk deposition velocity were examined, it was determined that 5-CBs, 6-CBs, and 8-CBs had higher deposition velocity in rainy period samples while 7-CBs and 8-CBs had more dominant characteristics in dry period samples.

Different parameters such as sampler type, atmospheric concentrations, meteorological parameters, sampling site characteristics, and sampling periods had effects on deposition mechanisms of the PCBs.

## Figures and Tables

**Table 1:** Measured meteorological data during the sampling period.

Number	Sampling period	Avg. temperature (°C)	Avg. wind speed (m/s)	Dominant wind direction	Avg. humidity (%)	Rain volume (*L*)
1	11.06–18.06.2008	24 ± 4	0.9 ± 1.0	WNW	48 ± 13	0
2	18.06–30.06.2008	27 ± 4	1.6 ± 1.0	NE	47 ± 13	0
3	30.06–16.07.2008	26 ± 4	1.7 ± 1.0	N	44 ± 11	0
4	16.07–01.08.2008	25 ± 4	1.5 ± 1.1	WNW	54 ± 14	0
5	01.08–15.08.2008	26 ± 4	1.7 ± 1.0	ENE	48 ± 11	0
6	15.08–01.09.2008	27 ± 4	1.5 ± 1.1	NE	55 ± 13	0
7	01.09–16.09.2008	24 ± 4	1.2 ± 0.9	WNW	53 ± 13	0.4
8	16.09–16.10.2008	17 ± 4	0.9 ± 1.1	WNW	72 ± 15	7
9	16.10–03.11.2008	16 ± 3	0.6 ± 0.7	NE	72 ± 12	0
10	03.11–17.11.2008	14 ± 3	0.7 ± 0.7	WNW	81 ± 10	0.25
11	17.11–01.12.2008	12 ± 5	2.3 ± 2.6	SE	69 ± 17	13.4
12	01.12–16.12.2008	11 ± 5	1.4 ± 1.1	E	64 ± 13	0.28
13	16.12–02.01.2009	5 ± 4	1.3 ± 1.0	ENE	77 ± 9	10.9
14	02.01–17.01.2009	5 ± 3	1.3 ± 0.9	E	68 ± 13	16.2
15	17.01–02.02.2009	10 ± 5	1.5 ± 1.7	ENE	66 ± 14	1.9
16	02.02–17.02.2009	10 ± 5	1.8 ± 1.8	WNW	71 ± 17	19.5
17	17.02–02.03.2009	5 ± 3	1.2 ± 1.0	ENE	76 ± 10	4
18	02.03–16.03.2009	10 ± 5	1.9 ± 2.0	WNW	71 ± 17	10
19	16.03–01.04.2009	9 ± 5	1.5 ± 1.6	WNW	66 ± 16	7
20	01.04–17.04.2009	12 ± 4	1.1 ± 0.9	WNW	72 ± 18	1
21	17.04–01.05.2009	14 ± 5	1.1 ± 1.0	WNW	67 ± 15	0.6
22	01.05–16.05.2009	17 ± 4	1.1 ± 1.1	WNW	62 ± 17	0
23	16.05–01.06.2009	20 ± 4	1.2 ± 1.0	WNW	57 ± 15	0
24	01.06–16.06.2009	24 ± 4	1.3 ± 1.1	WNW	57 ± 16	0

**Table 2:** Recovery efficiencies for the HVAS and BDS samplers.

Congeners	HVAS		WDDS		BDS
	Filter	PUF	Filter	Resin	
PCB#14	56.16 ± 14.79	70.22 ± 23.52	50.45 ± 25.83	47.16 ± 17.70	54.20 ± 11.46
PCB#65	62.50 ± 17.19	64.22 ± 18.33	67.47 ± 19.62	58.57 ± 18.78	77.25 ± 19.85
PCB#166	75.60 ± 23.20	77.44 ± 22.67	88.26 ± 26.55	72.30 ± 22.38	92.00 ± 17.74

**Table 3:** Atmospheric concentrations of PCBs at various locations.

Location		Period	n*	Concentration (pg/m^3^)	Reference
Paris, France	(Urban)	1989-1990	12	2000–6000	[3]
Cumbria, UK	(Coastal)	1996-1997	52	318	[27]
Athens, Greece	(Urban)	2000 July	38	344.9	[4]
	(Coastal)			181.1	
Thessaloniki, Greece	(Rural)	March 1999	6	0.5–29.2	[28]
	(Semirural)	October 1999		0.5–15.6	
Zagreb, Croatia	(Urban)	June 1999	20	8.2–968.6 (gas)	[29]
		February 2000		11.2–859.1 (particle)	
Madrid, Spain	(Urban)	February 1998	31	120–4300	[30]
		June 1998			
Stockholm, Sweden	(Urban)	1991–1996	63	3300–6800	[31]
Ansung, Korea	(Rural)	July 1999	38	6.13–71.9	[23]
		June 2000			
Venice Lagoon	(Urban)	August 2002	54	421 (gas)	[24]
		September 2002		11 (particle)	
Elm Road, England	(Semirural)	April 1999	12	252 (gas + particle)	[32]
		July 2000			
Chicago, USA	(Urban)	June 1995	50	1820 (gas)	[12]
		October 1995		90 (particle)	
Eordea, Greece	(Industrial)	January 2001	8	0.04–103 (particle)	[25]
		June 2001			
Yokohama, Japan	(Urban)	March 2002	12	62–250 (gas + particle)	[26]
		February 2003			
Uludag University Campus, Turkey	(Semirural)	July 2004	24	328 ± 284 (gas)	[33]
		May 2005		86 ± 128 (particle)	
Yavuzselim, Turkey	(Residential)	June 2008	83	320 ± 110 (gas)	This study
		June 2009		40 ± 30 (particle)	

**Table 4:** Dry and bulk deposition flux values obtained at different sampling site.

Sampling site	Period	n*	Flux (ng/(m^2^day))	Sampler type	Reference
Dry deposition					

Paris, France (Urban)	1989	12	79	Stainless-steel funnel	[3]
Sleeping Bear Dunes, MI, USA (Rural)	1993–1995	44	60	Mylar strips, Apiezon L. grease	[14]
Chicago, IL, USA (Urban)	June–October 1995	—	240	WSS	[34]
Bursa, Turkey (Suburban)	July 2004–May 2005	41	40.6 ± 40.6	WSS	[33]
Bursa, Turkey (Urban, Traffic site)	July 2004–May 2005	41	86.0 ± 97.4	WSS	[35]
Yavuzselim, Bursa, Turkey (Residential)	June 2008–June 2009	83	5.50 ± 2.50	WDDS	This study

Bulk deposition					

P. Marghera. Italy (Industrial)	1998-1999	—	7.3	Pyrex funnel	[37]
Valle Figeri Italy (Far-clean)	1998-1999	—	1.5	Pyrex funnel	[37]
Tsukuba. Japan (Semirural)	1997-1998	12	0.3	Stainless-steel vessel	[38]
Galveston Bay. USA (Coastal)	Feb. 1995–Aug. 1996	22	17.5	Wet deposition and calculation	[36]
BOID Bursa. TR (Urban/Industrial)	July 2004–May 2005	41	15.4 ± 14.3	Stainless-steel vessel	[16]
Gülbahçe, TR (Urban)	July 2004–May 2005	41	36.1 ± 21.3	Stainless-steel vessel	[35]
Yavuzselim, TR (Residential)	June 2008–June 2009	83	7.20 ± 3.50	Stainless-steel vessel	This study

*F = *V*_*d*_ × *C* (*V*_*d*_ = 0.2 cm/s).
